# A novel magnet based 3D printed marker wand as basis for repeated in-shoe multi segment foot analysis: a proof of concept

**DOI:** 10.1186/s13047-017-0220-7

**Published:** 2017-08-18

**Authors:** Maarten Eerdekens, Filip Staes, Thomas Pilkington, Kevin Deschamps

**Affiliations:** 1 Clinical Motion Analysis Laboratory, UZ Pellenberg, Weligerveld 1, 3212 Lubbeek, Belgium; 2 Department of Rehabilitation Sciences– Musculoskeletal Rehabilitation Research Group, KULeuven, Tervuursevest 101, B-3001 Leuven, Heverlee Belgium; 3 Fablab Leuven, Celestijnenlaan 300, 3001 Leuven, Heverlee Belgium; 4 Institut D’Enseignement Supérieur Parnasse Deux-Alice, Division of Podiatry, Bruxelles, Belgium; 5 Department of Podiatry, Artevelde University College, Ghent, Belgium

**Keywords:** Marker, Magnet, In-shoe, Multi-segment, Gait, 3D

## Abstract

**Background:**

Application of in-shoe multi-segment foot kinematic analyses currently faces a number of challenges, including: (i) the difficulty to apply regular markers onto the skin, (ii) the necessity for an adequate shoe which fits various foot morphologies and (iii) the need for adequate repeatability throughout a repeated measure condition. The aim of this study therefore was to design novel magnet based 3D printed markers for repeated in-shoe measurements while using accordingly adapted modified shoes for a specific multi-segment foot model.

**Methods:**

Multi-segment foot kinematics of ten participants were recorded and kinematics of hindfoot, midfoot and forefoot were calculated. Dynamic trials were conducted to check for intra and inter-session repeatability when combining novel markers and modified shoes in a repeated measures design. Intraclass correlation coefficients were calculated to determine reliability.

**Results:**

Both repeatability and reliability were proven to be good to excellent with maximum joint angle deviations of 1.11° for intra-session variability and 1.29° for same-day inter-session variability respectively and ICC values of >0.91.

**Conclusion:**

The novel markers can be reliably used in future research settings using in-shoe multi-segment foot kinematic analyses with multiple shod conditions.

## Background

Multi-segment foot kinematic analyses have gained enormous popularity in the last ten years since they provide a more detailed analysis of foot kinematics compared to single segment models [1, 2]. Until now, these multi-segment approaches have predominantly been used to assess barefoot foot kinematics [3–5]. Non-invasive in-shoe foot kinematics however, remain delicate to quantify as certain challenges arise: the difficulty to apply regular markers due to the fact that the skin is not directly accessible, the necessity for an adequate shoe which fits various foot morphologies and the need for adequate repeatability throughout a repeated measure condition. Several authors tried to overcome these challenges by introducing modified shoes that allow skin-mounted markers for in-shoe multi-segment foot analysis with and without foot orthoses [6–10]. Apart from shoe modification, other challenges occur when measuring in-shoe foot kinematics, as regular markers are complex to place on the foot when in shod condition. Bishop et al. have addressed this challenge by manufacturing a so-called two-part in-shoe marker wand [7]. Being an interesting development, our aim was to expand the applicability of this concept in order to provide a solution for future research investigating shod conditions/orthoses, as an optimal combination between modified shoes and feasible markers needs to be found for repeated measurement designs. The objective of the current study was to develop and assess reliability and repeatability of a thin and solid in-shoe marker wand, consisting of a baseplate and marker-unit, with user-friendly features that allow for repeated in-shoe multi-segment foot analyses using modified shoes.

## Methods

### Participants

Ten asymptomatic adults signed the informed consent and were selected on their anthropometric heterogeneity (Table 1). The Ethical committee of University Hospitals of Leuven (S57147) granted approval for the study. Exclusion criteria were any trauma possibly affecting normal gait. Sample size estimation (Power: 0.80, type-1 error: 0.05) for ICC values was conducted and suggested that 13 participants suffice for this reliability study when an expected reliability (ρ1) of 0.90 was applied [11].

**Table 1:** Demographic and anthropometric data of recruited participants

Participant/Gender/Age	Height (m)	Body mass (kg)	Shoe size (European)	FPI	Navicular height (mm)
1/M/36	1.80	72	43 1/2	5	42.1
2/M/28	1.79	90	44	2	62.1
3/F/25	1.76	71.8	42	2	51.3
4/F/24	1.59	63.6	38	6	47.9
5/M/24	1.80	65.5	43 1/2	3	58.6
6/M/21	1.70	62.2	43	3	59.8
7/M/49	1.69	67.8	43	4	42.6
8/M/23	1.84	85	43 1/2	6	41.3
9/F/39	1.65	61	42	7	39.8
10/F/39	1.73	59	42	7	34.2

*M* Male, *F* Female, *FPI* foot posture index, FPI ranges from −12 (highly supinated) to +10 (highly pronated)

### In-shoe wand marker and shoe modification

**Fig. 1:**
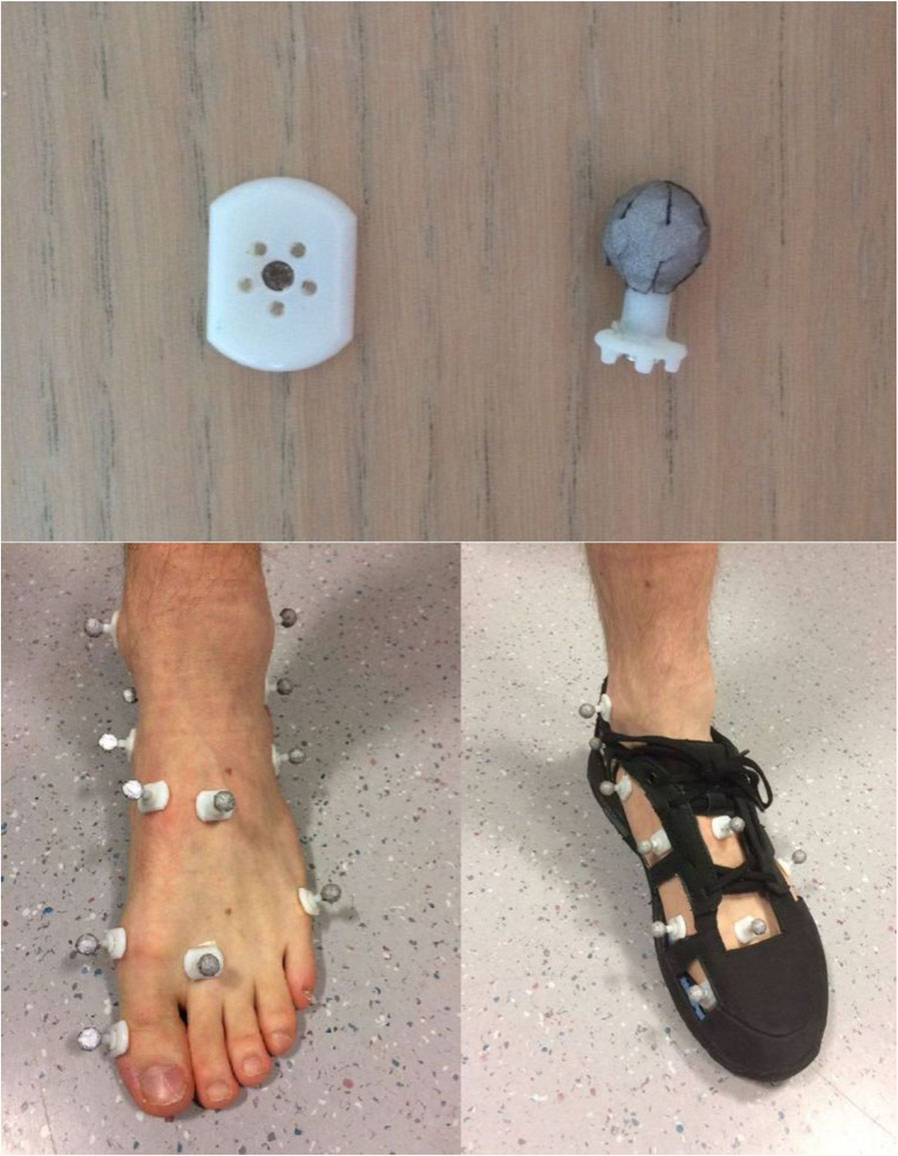
Novel magnet based in-shoe marker. Top: novel designed magnet based 3D printed marker, consisting of the baseplate (left) and the retro-reflective sphere unit (right). Bottom left: markers placed barefoot according to Rizzoli foot model. Bottom right: markers placed on foot within modified shoes for in-shoe multi segment foot analysis using the Rizzoli foot model

The newly developed magnet-based in-shoe wand marker consists of two units, where one unit functions as the baseplate and the second as a rod, containing a retro reflective sphere (Fig. 1). The baseplate (length 18.0 mm, width 13.0 mm and height 3.00 mm) has five 0.80 mm holes and one 5.00 mm hole, with a disc magnet glued in the latter. The five 0.80 mm holes are made with a same inter-hole distance around the magnet-hole and provide additional support to the rod. The marker rod (stick height 5.00 mm, stick width 4.00 mm, sphere diameter 9.00 mm and total height 15.5 mm) possesses a magnet fixed in the stick to connect with the magnet of the baseplate. As mentioned, two disc magnets are used, one for the baseplate (diameter 5 mm, height 2 mm) and one for the marker rod (diameter 3 mm, height 3 mm), both with a force equal to 5.1 and 2.84 Newton respectively (Supermagnete, Webcraft GmbH, Gottmadingen, Germany). Since accelerations during walking remain low, orthogonal forces applied onto the magnets are inferior to the magnet forces, causing that the marker wands stay fixated into their baseplates throughout measurements. The structure component of both units was 3D printed in VeroWhitePlus Polyjet (Stratasys, Eden Prairie, Minnesota, US) as this material has a tensile strength of 50–65 MPa, a polymerized density of 1.17 g/cm^3^ and flexural strength of 110 MPa, which compared to other commonly used materials is high.

A closed-toe shoe (Gel-odyssey WR, ASICS) with a black nubuck upper, a conventional laced fastening consisting of 5 eyelets, a rigid heel counter, a pitch of 1.4 cm, a single density midsole, moderate stiffness of the forefoot, a rubber outsole, a forefoot sole flexion point at the level of the metatarsal heads and a rigid midfoot sole (sagittal and frontal plane) was used in current study.

The shoe was modified by manually cutting eight windows into the upper of the shoe. In order to accommodate all foot types and individual anatomical differences, windows with a diagonal ranging from 3.5 cm to 8 cm were applied. Dimensions of each of these windows depended on the anatomical site of the foot, e.g. navicular tuberosity and base of first metatarsal bone are nearby each other. Therefore, one larger medial window was cut in order to more easily palpate both landmarks. Windows were also cut according to shoe size. By stitching the edges, the remaining materials surrounding the windows were subsequently reinforced. European shoe sizes of the modified shoes were unisex and ranged from 36 to 48 (Fig. 1).

### Data collection

After clinical exam assessing the Foot Posture Index (FPI) [12], participants were instrumented with baseplates of the novel in-shoe wand marker according to the Rizzoli foot model [5] and these were fixated onto the skin using double-sided tape (Scotch® 19 mm Double Sided, 3 M, Minnesota, US). First, 5 static trials were captured in sitting position while baseplates were applied and remained in situ for the duration of the testing session. In between the 5 static trials, the therapist removed and reapplied the rod to the baseplates to assess repeatability and robustness of the magnet fitting. A sitting position was used as this facilitated the participants in keeping their foot as still as possible in between trials. Next, participants entered in shod condition, rods were attached on their baseplates and a static trial (stance) was captured. Then, per three representative walking trials, rods were detached from the baseplates and participants were asked to remove the shoes while baseplates remained on the foot. After a 1-min break, participants re-entered in shod condition, rods were replaced on the baseplates and dynamic trial capturing restarted. This process was repeated up to three times so that a total of 9 trials were captured with replacing of the marker wands after trial 3 and 6. Each trial was time-normalized by interpolating the gait cycle into 100 frames.

Marker trajectories were captured using ten T-10 cameras and Nexus software (Vicon Motion Systems Ltd., Oxford Metrics, Oxford, England).

### Data analysis

A mean placement error for all five sitting static trials was measured to assess the robustness of the interference magnet fitting, using the standard deviations between each trial and compared these to the movement of a reference marker (lateral malleolus marker). Comparison with the reference marker was done in order to compensate for the involuntary movement of the foot and so solely assess marker placement errors of the novel magnet fitted marker wands.

Intra-session and same-day inter-session repeatability of all three walking sessions were calculated using standard deviations between trials within a session (intra-session) and between sessions (same-day inter-session). Standard deviations were assessed per frame of the gait cycle (100 frames) and a mean of all these standard deviations was calculated for easier comparisons. Ratio between inter- (nominator) and intra-session repeatability (denominator) was computed as well.

Intraclass correlation coefficients (ICC_3,1_) between three walking sessions were calculated for three variables (maximum peak value, minimum peak value and range of motion) during gait cycle in order to assess reliability. Additionally, standard error of the mean (SEM) was calculated for all joint angle rotations and 95% confidence intervals of absolute ROM (range of motion) of each joint angle and plane were assessed as well. Statistics were computed using MedCalc (MedCalc software BVBA, Ostend, Belgium) and Excel (Microsoft Corporation, Redmond, USA).

## Results

**Fig. 2:**
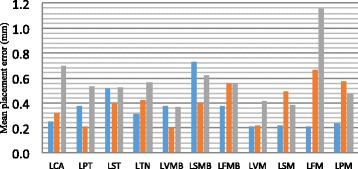
Mean marker placement errors. Mean placement errors of novel markers in sagittal plane (blue bar), frontal plane (orange bar) and transverse plane (grey bar). LCA: left calcaneus (reference marker); LPT: left peroneal tubercle; LST: left sustentaculum talus; LTN: left navicular tubercle; LVMB: left fifth metatarsal base; LSMB: left second metatarsal base; LFMB: left first metatarsal base; LVM: left fifth metatarsal head; LSM: left second metatarsal head; LFM: left first metatarsal head; LPM: left first phalanx

Mean placement error of all markers was 0.44 mm, with a maximum placement error of 1.16 mm and a minimum placement error of 0.2 mm (Fig. 2). Intra-session repeatability or natural gait variability varied between 0.30 degrees for calcaneal-midfoot joint in frontal plane (Y) and 1.11 degrees for shank-calcaneal joint in sagittal plane (X). For the inter-session repeatability, outcomes ranged between 0.41 degrees for calcaneal-midfoot joint in Y-plane and 1.29 degrees for midfoot-metatarsal joint in transverse plane (Z). Ratios mostly exceeded 1.00 (Table 2).

**Table 2:** Intraclass correlation coefficients of joint kinematics

Joint rotation angle:	ICC (95% CI)			SEM (95% CI)
	Max	Min	ROM	ROM
Sha - Cal DF/PF	0.980 (0.942–0.995)	0.978 (0.937–0.994)	0.966 (0.904–0.991)	0.59 (24.8–27.1)
Sha - Cal Inv/Ev	0.991 (0.975–0.998)	0.993 (0.979–0.998)	0.948 (0.853–0.986)	0.34 (9.24–10.6)
Sha - Cal Add/Abd	0.993 (0.980–0.998)	0.997 (0.992–0.999)	0.948 (0.835–0.986)	0.38 (10.7–12.2)
Cal - Met DF/PF	0.995 (0.986–0.999)	0.993 (0.980–0.998)	0.960 (0.883–0.989)	0.54 (12.3–14.4)
Cal - Met Inv/Ev	0.988 (0.964–0.996)	0.979 (0.940–0.994)	0.947 (0.840–0.986)	0.29 (5.70–6.90)
Cal - Met Add/Abd	0.947 (0.843–0.986)	0.986 (0.960–0.996)	0.944 (0.834–0.985)	0.69 (6.63–9.34)
Cal - Mid DF/PF	0.980 (0.943–0.995)	0.979 (0.940–0.994)	0.968 (0.908–0.991)	0.21 (6.30–7.13)
Cal - Mid Inv/Ev	0.979 (0.943–0.995)	0.934 (0.808–0.982)	0.792 (0.338–0.954)	0.12 (2.78–3.27)
Cal - Mid Add/Abd	0.992 (0.978–0.997)	0.987 (0.963–0.996)	0.917 (0.754–0.977)	0.23 (5.51–6.41)
Mid - Met PF/DF	0.994 (0.984–0.998)	0.995 (0.984–0.998)	0.968 (0.909–0.991)	0.49 (9.59–11.5)
Mid - Met Inv/Ev	0.981 (0.947–0.995)	0.977 (0.934–0.994)	0.942 (0.830–0.984)	0.24 (2.73–3.69)
Mid - Met Add/Abd	0.965 (0.897–0.991)	0.985 (0.955–0.996)	0.969 (0.908–0.992)	0.72 (5.43–8.27)
Hallux DF/PF	0.991 (0.975–0.998)	0.991 (0.974–0.998)	0.914 (0.757–0.977)	0.29 (6.09–7.24)

*Sha* shank, *Cal* calcaneus *Met* metatarsal, *Mid* midfoot, *PF* plantar flexion, *DF* dorsiflexion, Max highest peak value of joint angle during gait cycle; Min, lowest peak value of joint angle during gait cycle; ROM, Range of motion during gait cycle; ICC, intraclass correlation coefficients; SEM standard error of the mean; 95% CI, 95% confidence interval; Joint angle rotation in degrees

All ICCs were high (>0.91), except for calcaneus – midfoot rotation in transverse plane (0.792) (Table 3). SEMs remained below 0.8° for all joint rotation angles and 95% confidence interval, wherein the absolute range of motion is situated, was given as well (table 3).

**Table 3:** Intra – and inter-session variability of kinematic measurements

Joint rotation angle:	Intra-session variability			Inter-session variability			Ratio (inter/intra)		
	X	Y	Z	X	Y	Z			
Sha - Cal	1.11	0.88	0.75	0.87	0.82	0.77	0.79	0.94	1.03
Cal-Mid	0.53	0.30	0.56	0.70	0.41	0.85	1.32	1.37	1.50
Cal-Met	0.74	0.49	0.55	0.93	0.68	1.25	1.25	1.38	2.26
Mid-Met	0.60	0.37	0.59	0.83	0.81	1.29	1.40	2.16	2.19
Hallux (planar)	-	-	0.48	-	-	0.97			2.00

*Sha*, shank, *Cal* calcaneus, *Mid* midfoot, *Met* metatarsal, *X* sagittal plane, *Y* frontal plane, *Z* transverse plane

All results in degrees, except for ratio

## Discussion

The aim of this study was to design a novel magnet based, 3D printed marker wand for in-shoe multi-segment foot analysis using a repeated measurement design. To gain confidence in using these markers in research applications, repeatability and reliability was investigated for in-shoe walking trials.

Repeatedly taking on and off markers wands from the baseplates in static position generates low placement errors as the magnet fitting principle showed a solid robustness. This implies that once the baseplates are placed, marker wands can be taken on and off by any other therapist with the knowledge that outcomes will not vary significantly.

To assess repeatability when performing dynamic in-shoe multi-segment foot analysis with these new markers, we set up a design in which shoes ought to be taken on and off while baseplates remained on the foot during the whole measurements. Inter-session repeatability never exceeded 1.29 degrees (Table 3), meaning that this was the maximum difference in kinematic outcome values when measuring multiple shod conditions during one analysis. To correctly assess the influence of 1) natural gait variability and 2) repeatedly taking on and off the shoe, we calculated the ratio between these two test conditions. Putting on shoes multiple times influenced kinematic waveforms more than natural gait variability did. Though, absolute error values still remained very low as previously mentioned. This demonstrates the advantage of the magnet fitting principle, which insures near to identical positions of the marker wand, even when they ought to be taken on and off their baseplates. Also, changing shoes will not alter the baseplate position, which initially was considered as a risk since some of the baseplates are located on protrudes anatomical entities.

Reliability of measuring similar in-shoe foot kinematics in a repeated-measurements design tended to be very high as suggested by the ICC outcomes. Looking at existing research regarding reliability of placing regular markers, these novel markers score higher on average as suggested by ICC outcomes compared to these other studies [13, 14]. Although, discretion in comparison is warranted as methodology and experiment set-up may differ between studies.

Assessment of Foot Posture Index was conducted to check heterogeneity in foot morphologies between participants and appeared relatively high (cfr. 3.1). For this, modification of the shoes was done by cutting larger holes to overcome issues occurring when multiple participants with heterogeneous feet ought to be tested with identical shoe sizes. This, however, is also a first limitation to current study, as some shoe integrity may be lost due to this modification. Yet, it would be costly and time consuming to modify a pair of shoe per participant. Another known limitation is the fact that experienced testers are still required, since placing of the baseplate determines outcome values. The reference marker used to account for foot movement is not ideal, since this would imply that ankle and foot are rigid. Yet, we were not able to add another reference marker onto the foot, since this was impossible to standardize in this type of measurement. Also, due to circumstances, we were not able to conduct an inter rater reliability analysis, which is a limitation to current study. However, we believe that potential inter rater reliability in these novel markers will be alike to earlier research studying marker placement reliability, as placing of the baseplates is similar to regular marker placement. Last, additional research will be necessary to gain confidence for use in a clinical population and also in settings where running trials need to be captured, since forces on the foot will be greater and reliability of the robustness of the magnet-based link needs to be re-evaluated.

## Conclusion

The novel 3D–printed markers, consisting of a baseplate and a wand marker, are a reliable basis for future settings using in-shoe multi segment foot analysis when shoes aught to be taken on and off repeatedly. Results showed a solid robustness of the magnet fitting principle that allows for reliable kinematic outcomes in a repeated measurement design conducting in-shoe walking trials.

## Abbreviations

FPI: Foot Posture Index
ICC: Intraclass correlation coefficient
RO: Range of motion
SEM: Standard error of the mean
